# High Precision Signal Processing Algorithm for White Light Interferometry

**DOI:** 10.3390/s8127609

**Published:** 2008-12-01

**Authors:** Jeonggon Harrison Kim

**Affiliations:** Information and Communication Engineering Dept. Hansei University, 604-5 Dangjeong-dong Gunpo-city Kyunggi-do, Korea

**Keywords:** Phase delay estimation, white light interferometry, signal processing algorithm, fine tuning

## Abstract

A new signal processing algorithm for absolute temperature measurement using white light interferometry has been proposed and investigated theoretically. The proposed algorithm determines the phase delay of an interferometer with very high precision (≪ one fringe) by identifying the zero order fringe peak of cross-correlation of two fringe scans of white light interferometer. The algorithm features cross-correlation of interferometer fringe scans, hypothesis testing and fine tuning. The hypothesis test looks for a zero order fringe peak candidate about which the cross-correlation is symmetric minimizing the uncertainty of mis-identification. Fine tuning provides the proposed algorithm with high precision sub-sample resolution phase delay estimation capability. The shot noise limited performance of the proposed algorithm has been analyzed using computer simulations. Root-mean-square (RMS) phase error of the estimated zero order fringe peak has been calculated for the changes of three different parameters (*SNR*, fringe scan sample rate, coherence length of light source). Computer simulations showed that the proposed signal processing algorithm identified the zero order fringe peak with a miss rate of 3 × 10^-4^ at 31 dB *SNR* and the extrapolated miss rate at 35 dB was 3 × 10^-8^. Also, at 35 dB *SNR*, RMS phase error less than 10^-3^ fringe was obtained. The proposed signal processing algorithm uses a software approach that is potentially inexpensive, simple and fast.

## 1. Introduction

Although fiber optic interferometric sensors offer the possibility of performing measurements with very high sensitivity and resolution [1], they suffer from problems such as complex signal processing techniques, quadrature point stabilization, and uncertainty as to whether an increase or decrease in the value of measurands has occurred. In order to fully utilize the capability of fiber optic sensors, a different sensing principle termed as “white light interferometer” or “white light interferometry (WLI)” was developed [2].

**Figure 1.**
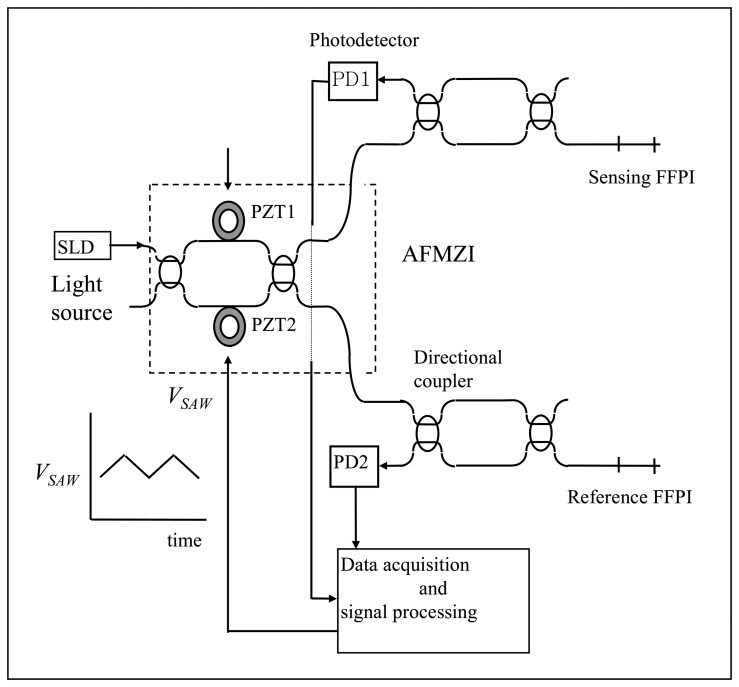
All Fiber White Light Interferometer.

From the beginning of this research, an all fiber white light interferometry (AFWLI) absolute temperature measurement system as shown in Figure 1 was selected as the application area of the proposed signal processing algorithm. White light interferometry departs from the conventional interferometry in that it uses a broadband light source. SLD in Figure 1 represents a superluminescent diode (SLD) used as a broadband light source and PD1, PD2 denote photodetectors 1 and 2, respectively. In the AFWLI two fiber Fabry-Perot interferometers (FFPI, sensing FFPI and reference FFPI in Figure 1) and a Mach-Zehnder Interferometer as a processing interferometer (scanning interferometer in Figure 1, hereafter termed as MZI), which has two piezoelectric transducers (hereafter termed as PZT) in its two arms, are connected in tandem. The sensing and the reference interferometer output signals from PD1 (Equation 1 for PD1) and PD2 (Equation 2 for PD2) are given respectively by:

(1) $$I_{S}(\Phi_{P})=1+\frac{1}{2}\exp\left\{-{\left[\frac{\Phi_{P}-\Phi_{S}}{\pi L_{C}/\lambda }\right]}^{2}\right\}\cos(\Phi_{P}-\Phi_{S})$$

(2) $$I_{R}(\Phi_{P})=1+\frac{1}{2}\exp\left\{-{\left[\frac{\Phi_{P}-\Phi_{R}}{\pi L_{C}/\lambda }\right]}^{2}\right\}\cos(\Phi_{P}-\Phi_{R})$$

where *L_C_* is the coherence length of light source, Φ*_P_* is the OPD (Optical Path-length Difference) between two arms of Mach-Zehnder processing interferometer and Φ*_S_*, Φ*_R_* are the round trip phase shifts for the sensing and reference FFPIs, respectively. In Equation 1 and Equation 2 it is assumed that light source has a Gaussian power spectrum. A constant d.c. voltage *V_DC_* (100∼150 volt, Figure 1) is applied to the PZT1 in one arm to coarsely match the OPD of the MZI to that of the sensing FFPI. And an alternating ramp voltage *V_SAW_* (Figure 1) is applied to PZT2 in the other arm to scan the processing interferometer so that OPD Φ*_P_* between two arms of MZI can be varied over a certain range.

**Figure 2.**
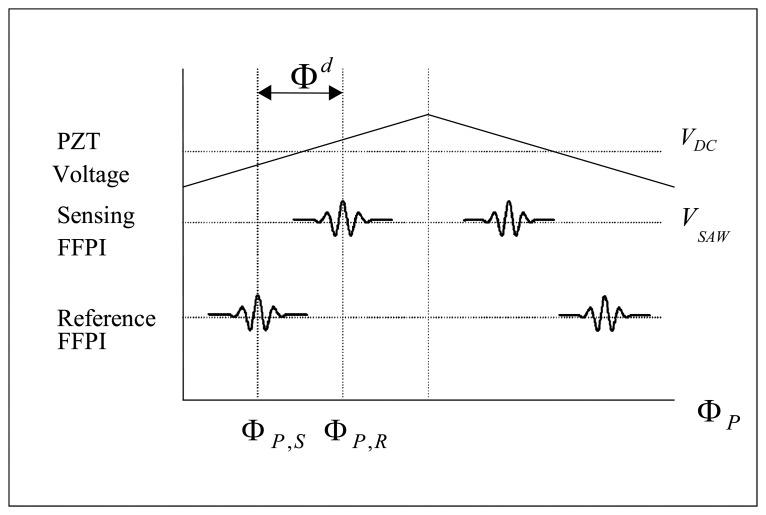
Output of sensing and reference FFPI from AFWLI.

This AFWLI for temperature measurement produces two fringe scans, one from the sensing FFPI and another one from the reference FFPI, as shown Figure 2. In AFWLI, the sensing FFPI is exposed to the temperature *T_S_* to be measured and the reference FFPI is protected from environmental disturbances but exposed to the known reference temperature *T_R_*. Now, assume that the known temperature of the sensing FFPI and the reference FFPI are *T_S_* and *T_R_*, respectively. When the phase Φ*_P_* of MZI is scanned and exactly matched to that of the sensing FFPI (the reference FFPI), a zero order fringe peak of the sensing FFPI (the reference FFPI) is produced at certain Φ*_P_* = Φ*_P_*_,_*_S_* (Φ*_P_* = Φ*_P_*_,_*_R_*), as shown in Figure 2. In Figure 2, Φ*_P_*_,_*_S_* (Φ*_P_*_,_*_R_*) denotes the phase of the processing interferometer producing the zero order fringe peak of the sensing FFPI (the reference FFPI). If we can identify the phase difference Φ*^d^* = Φ*_P,S_* – Φ*_P,R_* (this is possible by the proposed signal processing algorithm which will be shown later in this article) then Φ*^d^* is mapped to the temperature *T_S_* and absolute temperature measurement is possible. This problem has been known as “Time Delay Estimation (TDE) [3] (or Phase Delay Estimation)”. In this article, a new signal processing algorithm to estimate the phase delay Φ*^d^* of AFWLI is proposed. This article consists of five sections. Section 2 describes the previous related works for time delay estimation methods. In Section 3, a signal processing algorithm to measure the phase difference Φ*^d^* of AFWLI is proposed. In Section 4 the performance of the proposed signal processing algorithm has been demonstrated using computer simulations. Section 5 shows the comparison to the previous literature and Section 6 is the conclusion of this article.

## 2. Previous works

Two major classes of signal processing algorithms for WLI are the hardware approach and the software approach. Both approaches have a more or less “tracking zero order fringe peak” feature. Gerges proposed a hardware approach which locks the zero order fringe position of interferometer by a feedback loop [4]. An improvement of the sensitivity up to 1/240 fringe was claimed. To the author's best knowledge this method, while still dependent on the incremental characteristic of laser interferometry and not fully taking advantage of WLI's potential to identify the interference fringe, demonstrated the feasibility of locking and tracking the fringe peak for absolute measurement for the first time.

There are many software algorithms to estimate phase delay Φ*^d^* [3, 5]. Among many detection methods, the cross-correlation method dominates the field of phase delay estimation in practice due to its easier implementation [6]. Many other TDE methods are based on this algorithm. The cross-correlation method cross-correlates the two fringe signals *i_S_*(*n*) and *i_R_*(*n*), which are sampled versions of *I_S_*(Φ*_P_*) and *I_R_*(Φ*_P_*) respectively, into *i*(*n*) and considers the sample number argument *n*=*n_d_* that corresponds to the maximum peak in cross-correlation *i*(*n*) as the estimated time delay [7].

**Figure 3.**
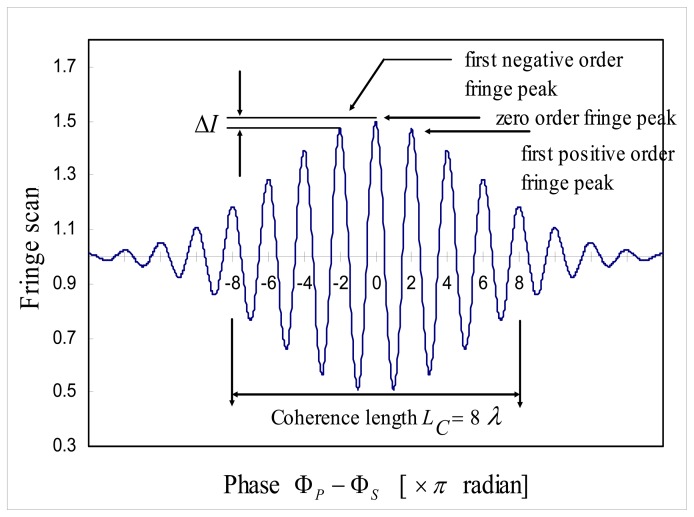
Output of white light interferometer (*I_S_* in Equation 1 or *I_R_* in Equation 2).

While WLI has the potential to identify the interference fringe order from the output pattern of an interferometer [8], it is difficult to distinguish the zero order fringe peak from its adjacent first order fringe peaks when noise is present in the interferometer output, as shown in Figure 3. From Equations 1 and 2, the amplitude difference Δ*I* between the zero order fringe peak and the first order fringe peaks is represented as:

(3a) $$\Delta I=\frac{1}{2}\left(1-\exp\left[-{\left(\frac{2}{L_{C}/\lambda }\right)}^{2}\right]\right)$$

Clearly, if a system has a noise level which is equal or greater than Δ*I* the zero order fringe peak cannot be identified directly, simply through inspection of its amplitude. If the normalized zero order fringe peak value is defined as unit signal, a minimum signal-to-noise ratio *SNR*_min_ required to identify the zero order fringe peak [9] is given by

(3b) $${\text{SNR}}_{\min}=-20\log\left[\frac{\Delta I}{\left(1/2\right)}\right]=-20\log\left\{1-\exp\left[-{\left(\frac{2}{L_{C}/\lambda }\right)}^{2}\right]\right\}$$

Representative values of the coherence length of different light sources like white light lamp, light emitting diode (LED), superluminescent diode (SLD), are about 10, 20, and 40 in terms of optical fringes. The *SNR*_min_ required to identify the zero order fringe peak by amplitude difference Δ*I* is given from Equation 3 as 28 dB, 40 dB, and 52 dB respectively [9]. One disadvantage related to cross-correlation is the broadening of envelope (or peak) (hereafter termed as envelope) from *L_C_* to $\sqrt{2}L_{C}$ due to the cross-correlation of two almost identical Gaussian envelope signals [17]. This results in higher *SNR*_min_ in Equation 3 requiring 6 dB more than before cross correlation. Then, with this broadening, the attainable resolution is often not better than one fringe and a precise peak location may be somewhat questionable. This difficulty has inhibited the application of fiber optic sensors using WLI, for example, absolute OPD determination [9].

Additionally, once the zero order fringe peak is identified, then for a more accurate sub-sample resolution time delay estimation we will have to use interpolation which is possible by either quadratic interpolation in time domain or frequency domain zero-padding [10, 11]. But quadratic interpolation uses three cross-correlation values centered at the estimated peak of cross-correlation. This method has a shortcoming of bias caused by time domain sample rate [12, 13] and the difference between true peak shape and the fitted quadratic polynomial. In frequency domain zero-padding, the number of zeros are padded in the middle of Fourier Transform of cross-correlation *i*(*n*). Notice that zero-padding in frequency domain increases the discrete frequency by a certain factor which eventually results in time-domain interpolation in cross-correlation signal *i*(*n*). Zero-padding in frequency domain is a useful tool to improve the peak location accuracy, but it increases the computational complexity [14] and storage requirement associated with inverse Fast Fourier Transform (FFT) operations [15].

Notwithstanding the above mentioned shortcomings, cross-correlation is a still useful tool for time delay estimation as shown that cross-correlation with no pre-filtering is an optimal maximum likelihood estimator to estimate the time delay between two similar signals if the noise processes *w_R_*(*n*), *w_S_* (*n*) of signal *i_R_* (*n*), *i_S_* (*n*) are white noises and at least one of signals has high signal-to-noise ratio greater than 10 dB [16].

The outcome of the time delay estimation depends on the combined performance of coarse estimation (zero order fringe peak identification) and sub-sample resolution estimation of time delay. In this article, a new signal processing algorithm which can accurately identify the zero order fringe peak of cross-correlation *i*(*n*) of two fringe scan output signals of a WLI is proposed. This algorithm still uses a cross-correlation technique taking advantage of simple implementation. But this algorithm combines the hypothesis test as a coarse estimation to reduce the possibility of mis-identification of peak with fine tuning algorithm as a sub-sample resolution peak estimation to overcome the shortcomings of quadratic interpolation or frequency domain zero-padding.

The proposed signal processing algorithm uses a software approach, which is potentially inexpensive, simple and fast. And, this proposed signal processing algorithm has a low peak mis-identification rate of 3 × 10-^4^ at 31 dB *SNR* and has a high precision fine tuning capability down to 5 × 10-^4^ fringe as will be shown from the computer simulation results.

## 3. Proposed Signal Processing Algorithm

The proposed signal processing algorithm consists of five steps applied to sampled signal of WLI fringe scans. They are:

1) Normalization and cross-correlation
2) Peak and zero crossing detection
3) Matched filtering
4) Hypothesis test
5) Fine Tuning

Each procedure is explained briefly below.

### 3.1. Normalization and cross-correlation

As a preliminary procedure, the output of photodetector signals *I_S_* (Φ*_P_*) and *I_R_* (Φ*_P_*) are sampled and normalized respectively into fringe scan *i_S_* (*n*) and *i_R_*(*n*):

(4) $$i_{S}(n)=\exp\left\{-{\left[\frac{2\pi (n-n_{S})/f_{S}}{\pi L_{C}/\lambda }\right]}^{2}\right\}\cos\{2\pi (n-n_{S})/f_{S}\}+w_{S}(n)$$

and:

(5) $$i_{R}(n)=\exp\left\{-{\left[\frac{2\pi (n-n_{R})/f_{S}}{\pi L_{C}/\lambda }\right]}^{2}\right\}\cos\{2\pi (n-n_{R})/f_{S}\}+w_{R}(n)$$

where *n_S_* is zero order fringe peak sample number for sensing FFPI, *n_R_* is zero order fringe peak sample number for reference FFPI, *f_S_* is the sample rate in samples per fringe (or samples/fringe). A normalization procedure is carried out by removing the d.c. component (constant “1” in Equation 1 and Equation 2) of each fringe scan. *w_S_*(*n*) and *w_R_*(*n*) are the white noise related to the fringe scan *i_S_*(*n*) and *i_R_*(*n*) respectively with zero mean and variance $\sigma_{w}^{2}$. The phase delay between the sensing FFPI and the reference FFPI is defined in terms of samples as:

(6) $$n_{d}=n_{S}-n_{R}$$

After normalization, *i_S_*(*n*) and *i_R_*(*n*) are cross-correlated into *i*(*n*) and normalized again. Cross-correlation *i*(*n*) can be expressed in mathematical form with its zero order fringe peak *p_0_* at *n*=*n_d_* as shown in Equation 7.

(7) $$\begin{array}{c} i(n)=\sum_{k=-\infty }^{\infty }i_{R}(k)i_{S}(n+k)\\ =\exp\left\{-{\left[\frac{2\pi (n-n_{d})/f_{S}}{\pi L_{C,\text{eff}}/\lambda }\right]}^{2}\right\}\cos\{2\pi (n-n_{d})/f_{S}\}+w(n) \end{array}$$

In Equation 7 the effective coherence length *L_C_*_,_*_eff_* is given as $\sqrt{2}L_{C}$ [17] and *w*(*n*) is a white noise of *i*(*n*). Exponential term in Equation 7 is termed as “the envelope” of cross-correlation *i*(*n*). This cross-correlation improves *SNR* at zero order fringe peak up to 14 dB [17]. One disadvantage related to cross-correlation is the broadening of peak from *L_C_* to $\sqrt{2}L_{C}$ due to the cross-correlation of two (almost) identical Gaussian envelope signals [17]. For example, this results in higher *SNR*_min_ in Equation 6 requiring 6 [dB] more than before cross-correlation when *L_C_*=26λ but this will be compensated by 14 dB *SNR* improvement at the zero order fringe peak [17]. After cross-correlation, the task of the signal processing algorithm becomes to find a zero order fringe peak *p*_0_=*n_d_* (global maximum) of cross-correlation *i*(*n*) correctly.

### 3.2. Peak and Zero-crossing Detection

**Figure 4.**
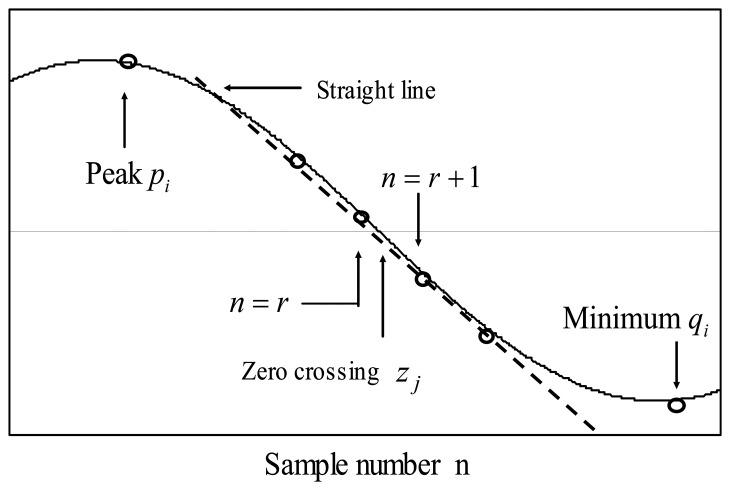
Linear Interpolation and zero crossing.

At this stage, all the peaks *p_i_*, all the minima *q_i_* and all the zero crossings *z_i_* of the cross-correlation *i*(*n*) are detected and registered. Every peak is labeled as *pi* where its value *p*_0_=*n_d_* is zero order fringe peak position, *p*_-1_ is negative first order fringe peak position, *p*_1_ is positive first order fringe peak position in terms of sample number and so on. Also every zero crossing between peak *p_i_* and minimum *q_i_* is detected and labeled as *z_j_* where *j*=1,2,3…in terms of sample number. Then zero crossing position *z_j_* with sub-sample resolution is calculated [9] by using interpolation formula as shown in Equation 8, Equation 9 and Figure 4.

(8) $$z_{j}=r+\frac{i(r)}{i(r)-i(r+1)}\text{for}|i(r)|>|i(r+1)|$$

and

(9) $$z_{j}=r+1-\frac{i(r+1)}{i(r+1)-i(r)}\text{for}|i(r)|<|i(r+1)|$$

Linear interpolation is the reasonable method because the cosine function crosses the zero essentially as a straight line as shown in Figure 4.

Table 1 shows one example of peak and zero crossing table. Note that in this table peak position is a integer number but zero crossing is not necessarily a integer number due to the interpolation calculation shown in Equation 8 and Equation 9.

**Figure 5.**
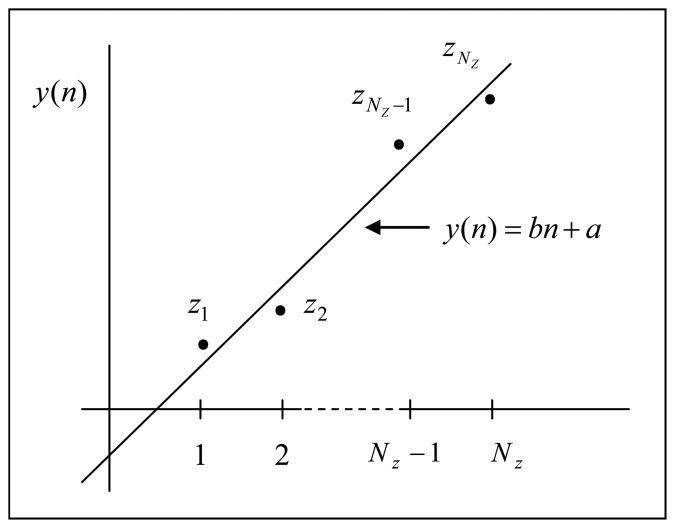
Least Square Fit of zero crossing positions.

Then the zero crossing period *b* is calculated by fitting all the *z_j_* into the form of:

(10a) $$\begin{array}{cc} y(n)=bn+a & \text{for n}=1,2,3,\ldots N_{Z} \end{array}$$

by applying least square fitting to the distance between all pairs of zero (*z_j_* and *z_j_*_+_*_1_*) as shown Figure 5 where *N_Z_* is the total number of zero crossings in *i*(*n*). In Figure 5 the slope *b* of the *y* (*n*) is the estimated number of samples inside half fringe (or half the sample rate) and the estimated sample rate is given by:

(10b) $${\hat{f}}_{S}=[2b]$$

where [2*b*] is the closest integer number of 2*b*.

### 3.3. Matched Filtering

A matched filter is the optimum filter to maximize the *SNR* of signal out of the matched filter in the presence of additive stochastic noise at input signal [18]. Consider the case where the input signal is *s*(*t*) and *n*(*t*) is the white noise with zero mean, variance $\sigma_{N}^{2}$ related to *s*(*t*). In this case, matched filter theory states that the maximum *SNR* at the output will occur when the matched filter has an impulse response *h*(*t*) = *s*(*t_0_*-*t*) that is equal to the time-reversed version of the signal waveform *s*(*t*) to which matched filter is matched and time-delayed by certain *t*_0_ [second]. When input signal *s*(*t*) is time-limited signal existing only when *0*< *t* <*T* then the matched filter is defined by [18]:

(11a) h(t)=s(T−t)

Note that the output *s*_0_(*t*) of the matched filter, which is the convolution of *s*(*t*) and *h*(*t*)=*s*(*T*-*t*), is given by:

(11b) $$s_{o}(t)=h(t)*s(t)=\int_{0}^{\infty }h(\lambda )s(t-\lambda )d\lambda =\int_{0}^{\infty }s(T-\lambda )s(t-\lambda )d\lambda$$

In Equation 11b *s*_0_*(t)* is the cross-correlation of the time-reversed version of input signal *s*(*t*) (i.e. *s*(-*t*)) with *h* (*t*)=*s*(*T*-*t*), which is denoted as *R_s_*_(_*_-t_*_)_*_h_*_(_*_t_*_)_(*t*). But *s*(*-t*) and *h*(*t*) are identical two waveforms with time distance *T* separated and *s*_0_(*t*)=*R_s_*_(_*_-t_*_)_*_h_*_(_*_t_*_)_(*t*) becomes *R_ss_*(*t-T*) when *R_ss_*(*t*) denotes the autocorrelation *R_ss_*(*t*) of input signal *s*(*t*). Then *s*_0_(*t*) has its maximum value when *t*=*T* and this maximum value is same as the *R_ss_*(0) of autocorrelation *R_ss_*(*t*) of input signal *s*(*t*).

Thus if we process a signal-plus-noise with a matched filter, the largest peak outputs due to the signal will correspond to *R_ss_*(0) and the *SNR* [18] of the largest peak output of matched filter is given by:

(12) $${\text{SNR}}_{\text{output of matched filter}}=\frac{\sqrt{R_{SS}(0)}}{\sigma_{N}}$$

If we define the *SNR* of input signal *s(t)* as the ratio between the largest value *â* of input signal *s*(*t*) and σ*_N_*, then *SNR* improvement due to the matched filtering is given as:

(13) $${\text{SNR}}_{\text{imp},\text{matched}}=\frac{\sqrt{R_{SS}(0)}}{\sigma_{N}}/\frac{\hat{a}}{\sigma_{N}}=\frac{\sqrt{R_{SS}(0)}}{\hat{a}}$$

At this stage, following the concept of matched filter, only one fringe of cross-correlation signal *i* (*n*) (i.e. signal between *i*(*p_i_*) and *i*(*p_i_*_+_*_1_*) of *i* (*n*)) is considered as the signal to be matched and one period of cosine function, $\cos\left(\frac{2\pi n}{{\hat{f}}_{S}}\right)$ is considered as the matched filter, *i_M_*(*n*).

(14) $$\begin{array}{cc} i_{M}(n)=\cos\left(\frac{2\pi n}{{\hat{f}}_{S}}\right) & n=0,1,\ldots {\hat{f}}_{S} \end{array}$$

Then, for a given one fringe of cross-correlation signal *i*(*n*), signal out of the matched filter is maximized when the time delay between two signals is *f̂_S_* and maximum value is calculated as in Equation 15.

(15) $$J_{i}=\sum_{n=p_{i}}^{p_{i+1}}i(n)i_{M}(n)=\sum_{n=p_{i}}^{p_{i+1}}i(n)\cos\left(\frac{2\pi n}{{\hat{f}}_{S}}\right)$$

for given *J_i_*'*s* (*i*=0, ±1, ±2,…). The envelope function of *i*(*n*) is slowly changing in the vicinity of the zero order fringe peak of *i*(*n*) and Equation 15 can be rewritten as:

(16a) $$J_{i}=\sum_{n=p_{i}}^{p_{i+1}}i(n)\cos\left(\frac{2\pi n}{{\hat{f}}_{S}}\right)≅\sum_{n=p_{i}}^{p_{i+1}}\left\{h_{i}\cos\left(\frac{2\pi n}{f_{S}}\right)\right\}\cos\left(\frac{2\pi n}{{\hat{f}}_{S}}\right)$$

where *h_i_* in Equation 16 is the average value of exponential envelope function between *p_i_* and *p_i_*_+_*_1_* as shown in Equation 16b.

(16b) $$h_{i}=\frac{i(p_{i})+i(p_{i+1})}{2}$$

For the case of a zero order fringe peak *i*(*p*_0_) of *i*(*n*), average value *h*_0_ of envelope between *p*_0_ and *p*_1_ is approximately “1” because *i* (*p*_0_) =1 and *i* (*p*_0_) ≈ *i*(*p*_1_). Then, Equation 13 becomes:

(17) $${\text{SNR}}_{\text{imp},\text{matched}}=\frac{\sqrt{R_{SS}(0)}}{\hat{a}}=\frac{\sqrt{\sum_{n=0}^{{\hat{f}}_{S}}\cos^{2}\left(\frac{2\pi n}{{\hat{f}}_{S}}\right)}}{1}=\sqrt{\pi }$$

and *SNR* of *i*(*p*_0_) is improved by the factor of:

(18) $$20\log(\sqrt{\pi })\approx 5[\text{dB}]$$

In the above Equation 16a any *J_i_* value is the weighted integration of one fringe between two peaks of *i*(*n*) and also can be considered as maximum value out of matched filtering which has a improved *SNR* over *i*(*p_i_*) as shown Equation 13. So, *J_i_* values will be used in the hypothesis test instead of peak value *i*(*p_i_*) of *i*(*n*) as will be shown later (see Section 3.4.). One useful property of *J_i_* is *J*_0_=*J_-1_*, *J*_1_=*J*_-2_ …(*J*_i_=*J*_-__i-1_) due to the even symmetric property of *i* (*n*) about *n*=*p*_0_= *n_d_*.

### 3.4. Hypothesis Test

In hypothesis test, signal processing algorithm chooses the nine biggest peaks of *i*(*n*) as zero order fringe peak candidates. Ideally, *p_j_*'s of *j*=0, ±1, ±2, ±3, ±4 are selected. Hypothesis test presumes that each candidate peak is the zero order fringe peak and calculates the parameter *g*(*p_j_*):

(18) $$\begin{array}{cc} g(p_{j})=\sum_{i=0}^{\infty }\left(J_{j+i}-J_{j-i-1}\right)=\sum_{i=0}^{\infty }d(j,i) & \text{for}\;j=0,\pm 1,\pm 2,\pm 3,\pm 4 \end{array}$$

where *j* of notation *d*(*j,i*) corresponds to the candidate peak *p_j_* on the hypothesis test and *i* is the distance from the zero order fringe peak candidate in terms of fringe. Then *g*(*p*_0_) is expressed as:

(19) $$\begin{array}{c} g(p_{0})=d(0,0)+d(0,1)+d(0,2)+\ldots \\ =(J_{0}-J_{-1})+(J_{1}-J_{-2})+(J_{2}-J_{-3})+\ldots \end{array}$$

**Figure 7.**
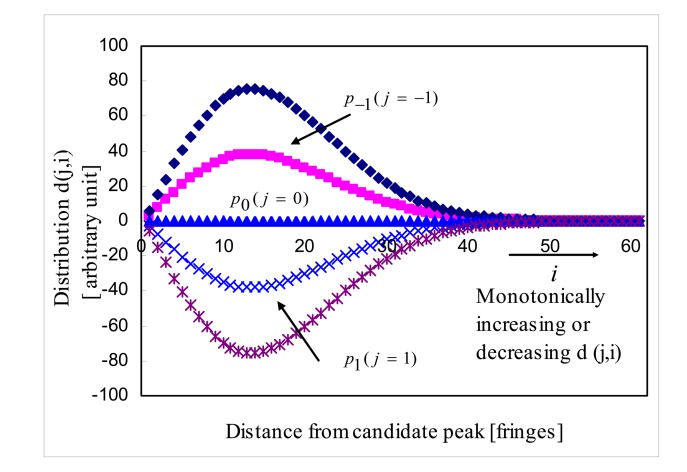
Distribution *d*(*j,i*) of noise-free cross-correlation.

Ideally all the values of *d*(*j*=0,*i*) for the zero order fringe peak *p*_0_ is zero (Figure 7) due to the symmetric property of *J_i_*= *J_-i-1_* and the zero order fringe peak candidate producing |*g*(*p_j_*)| =0 is announced as the estimated zero order fringe peak *p̂*_0_. But, practically the zero order fringe peak candidate producing minimum |*g*(*p_j_*)| is announced as the estimated zero order fringe peak *p̂*_0_. Note that ideally zero order fringe peak *p*_0_ happens at *n*= *n_d_*_,_ first positive order fringe peak *p*_1_ at *n*= *n_d_* + *f_S_* and so on.

### 3.5. Fine Tuning

**Figure 8.**
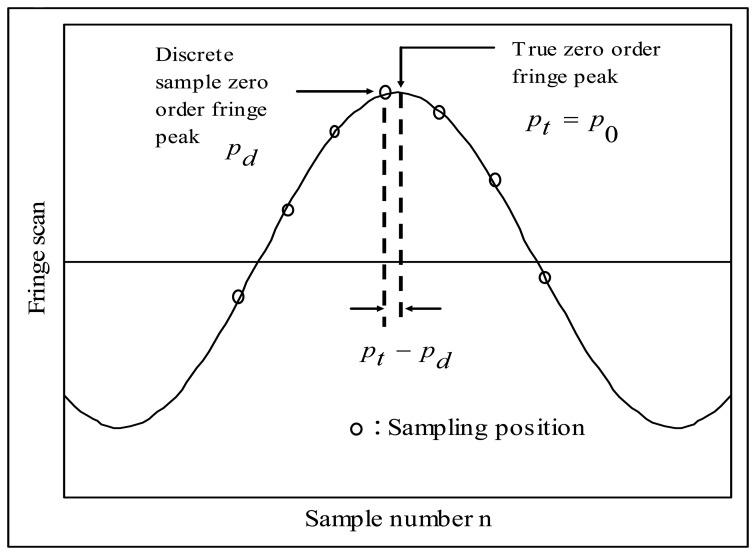
Illustration for true zero order fringe peak and discrete sample zero order fringe peak of *i*(*n*).

Figure 8 shows the vicinity of zero order fringe peak of cross-correlation *i*(*n*). From Figure 8 it is clear that the discrete sample zero order fringe peak position *p_d_* of the cross-correlation *i*(*n*) identified by the hypothesis test is not necessarily same as the “true zero order fringe peak position”, *p_t_* and the goal of fine tuning algorithm is to find the distance between *p_d_* and *p_t_*.

First, fine tuning algorithm assumes that the cross-correlation *i* (*n*) is represented as:

(20) $$i(n)=\exp\left\{-{\left[\frac{2\pi \left(n-p_{t}\right)/f_{S}}{\pi L_{C,\text{eff}}/\lambda }\right]}^{2}\right\}\cos\left\{\frac{2\pi (n-p_{t})}{f_{S}}\right\}$$

Note that *p_t_* in Equation 20 is not necessarily a integer sample number. Also, fine tuning algorithm generates test cross-correlation, *i_test_*(*n*) defined by:

(21) $$\begin{array}{c} i_{\text{test}}(n)=\exp\left\{-{\left[\frac{2\pi (n-p_{d})/{\hat{f}}_{S}}{\pi {\hat{L}}_{C,\text{eff}}/\lambda }\right]}^{2}\right\}\cos\left\{\frac{2\pi (n-p_{d})}{{\hat{f}}_{S}}\right\}\\ =\exp\left\{-{\left[\frac{2\pi (n-n_{d})/{\hat{f}}_{S}}{\pi {\hat{L}}_{C,\text{eff}}/\lambda }\right]}^{2}\right\}\cos\left\{\frac{2\pi (n-n_{d})}{{\hat{f}}_{S}}\right\} \end{array}$$

In Equation 21 it is assumed that the discrete sample zero order fringe peak position *p_d_* happens ideally at *n*= *n_d_* and also that the approximate value of the coherence length of the light source, *L̂_C_* is known priori to us. This is possible by counting the number of fringes within full width at which the intensity of interferogram using a particular light source decreases down to *e^-^*^1^ of its maximum value. And value of *f̂_S_* is given from *f̂_S_* = [2b] as shown in Section 3.2.

Then, the distance between *p_d_* and *p_t_* can be found by calculating *J(M)* for varying integer number M:

(22) $$\underset{-\infty <M<\infty }{J(M)}=\sum_{n=-\infty }^{\infty }i(n)i_{\text{test}}(n-M\Delta n)$$

where *i_test_*(*n*-*M*Δ*n*) is a computer-generated test cross-correlation with its zero order fringe peak at *p_d_* +*M*Δ*n* and Δ*n* is the desired fine tuning resolution in sample.

(23) $$\Delta n=\frac{{\hat{f}}_{S}}{N_{\text{sub}}}[\text{sample}]$$

In Equation 23 *N_sub_* is the number of sub-divisions in one fringe (the desired fine tuning resolution in fringe). Then *J*(*M*) becomes:

(24) $$\begin{array}{c} \underset{-\infty <M<\infty }{J(M)}=\sum_{n=-\infty }^{\infty }\exp\left\{-{\left[\frac{2\pi (n-p_{t})/f_{S}}{\pi L_{C,\text{eff}}/\lambda }\right]}^{2}\right\}\cos\left\{\frac{2\pi (n-p_{t})}{f_{S}}\right\}\times \\ \exp\left\{-{\left[\frac{2\pi (n-n_{d}-M\Delta n)/{\hat{f}}_{S}}{\pi {\hat{L}}_{C.\text{eff}}/\lambda }\right]}^{2}\right\}\cos\left\{\frac{2\pi (n-n_{d}-M\Delta n)}{{\hat{f}}_{S}}\right\} \end{array}$$

Interpretation of Equation 24 is that fine tuning algorithm generates its own test cross-correlation, *i_test_*(*n*) with the true zero order fringe peak positioned at *n_d_* +*M*Δ*n* and calculate *J*(*M*) for varying M. Then *J*(*M*) has a peak value at the certain *M_f_* which makes *M_f_*Δ*n* ≈ *p_t_*-*n_d_* because the best similarity between *i* (*n*) and *i_test_* (*n*) is attained when *n_d_* +*M_f_*Δ*n* ≈ *p_t_* Then, fine tuning algorithm announces (*n_d_* +*M_f_*Δ*n*) as the estimated true zero order fringe peak location *p̂_t_*.

Calculating *J*(*M*) for the range of - ∞ ≤ M ≤ + ∞ takes many calculations and is time-consuming. The true zero order fringe peak *p_t_* normally is within several samples of the discrete sample zero order fringe peak *p_d_*. Especially, when *SNR* of the fringe scans is high enough, the true zero order fringe peak *p_t_* is ideally within the half sample of the discrete zero order fringe peak *p_d_*. So, for the above case *J*(*M*) is calculated only for the range of $\left[-\frac{N_{\text{sub}}/{\hat{f}}_{S}}{2}\right]\leq M\leq \left[\frac{N_{\text{sub}}/{\hat{f}}_{S}}{2}\right]$ for faster signal processing and Equation 24 can be expressed as shown Equation 25 where $\left[\frac{N_{\text{sub}}/{\hat{f}}_{S}}{2}\right]$ is the closest integer number of $\frac{N_{\text{sub}}/{\hat{f}}_{S}}{2}$.

(25) $$\begin{array}{c} \underset{-\left[\frac{N_{\text{sub}}/{\hat{f}}_{S}}{2}\right]<M<\left[\frac{N_{\text{sub}}/{\hat{f}}_{S}}{2}\right]}{J(M)}=\sum_{n=-\infty }^{\infty }\exp\left\{-{\left[\frac{2\pi (n-p_{t})/f_{S}}{\pi L_{C,\text{eff}}/\lambda }\right]}^{2}\right\}\cos\left\{\frac{2\pi (n-p_{t})}{f_{S}}\right\}\times \\ \exp\left\{-{\left[\frac{2\pi (n-n_{d}-M\Delta n)/{\hat{f}}_{S}}{\pi {\hat{L}}_{C,\text{eff}}/\lambda }\right]}^{2}\right\}\cos\left\{\frac{2\pi (n-n_{d}-M\Delta n)}{{\hat{f}}_{S}}\right\} \end{array}$$

Additionally it must be emphasized that the principle of fine tuning in Equation (24) and Equation (25) can be used for matched filtering in order to obtain the best similarity between the signal inside the one fringe of cross-correlation (signal between any peak *i*(*p_i_*) and *i*(*p_i_*_+1_)) and the matched filter *i_M_*(*n*) and to maximize the *SNR* improvement at peaks by matching the phase between the discrete sample *i*-th order fringe peak *i*(*p_i_*) and the very first value of the matched filer *i_M_*(*n*).

## 4. Computer Simulations

The proposed signal processing algorithm was verified using computer simulations. To see the shot-noise limited performance of the proposed signal processing algorithm, the normalized AFWLI fringe scans, *i_S_*(*n*) and *i_R_*(*n*) were computer-generated using Equation 4 and Equation 5 respectively and shot noise was added to the AFWLI fringe scans, *i_S_*(*n*) and *i_R_*(*n*) instead of white noise. In the computer simulations the zero order fringe peak position *p_0,S_* (and *p_0_*_,_*_R_*) for *i_S_*(*n*) (and *i_R_*(*n*)) were randomly selected as real number. *i_S_*(*n*) and *i_R_*(*n*) were cross-correlated into *i*(*n*). Then, *p_t_* is calculated as (*p_0,S_*-*p_0R_*) and zero order fringe peak *p_d_* = *n_d_* of *i*(*n*) becomes the integer part of *p_t_*. The coherence length of *i_S_*(*n*) and *i_R_*(*n*) were chosen as *L_C_*=26*λ* to simulate the coherence length of the commercially available SLD like OKI OE350S from Oki semiconductor. For all the computer simulations presented in this article parameters are fixed as follows, unless the specified parameter is varied for a certain range and circumflex notation ∧ on the top of the parameter denotes the estimated value of that particular parameter calculated by the proposed signal processing algorithm.

- Sample rate *f_S_* =16 [samples/fringe]
- *SNR*=30 dB
- Effective Coherence length $L_{C,\text{eff}}=\sqrt{2}\times 26\lambda \approx 36\lambda$
- Size of fine tuning step Δ*n*=1/1000 [fringe]

### 4.1. Simulation: Miss rate simulation

In the first simulation miss rate (misidentification rate) of the proposed signal processing algorithm was tested at different shot noise levels. The *SNR* was varied from 1 dB to 28 dB with 1 dB separation and a set of 10000 simulations was carried out at different *SNR*. When the position difference between the estimated zero order fringe peak *p̂_d_* and the computer-generated zero order fringe peak *p_d_* is bigger than half fringe (8 samples), the zero order fringe peak is considered to have been misidentified. Table 2 shows the miss rate of the proposed signal processing algorithm and miss rate is defined as the ratio of number of miss to 10000.

**Figure 9.**
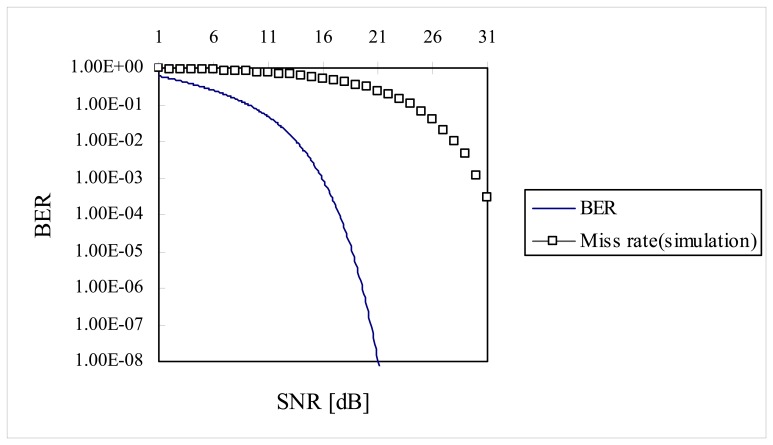
Comparison between miss rate and BER.

In Figure 9, miss rate was plotted along with Bit Error Rate (BER) of binary fiber optic communication system [19]. In Figure 9 (or Table 2) we have a rule of thumb that every dB improvement in *SNR* (over the range of 26 ∼ 31 dB) produces approximately one order of magnitude improvement in error rate. This kind of behavior is also the case for binary fiber optic communication system (two orders of magnitude improvement in error rate for the binary fiber optic communication system).

**Figure 10.**
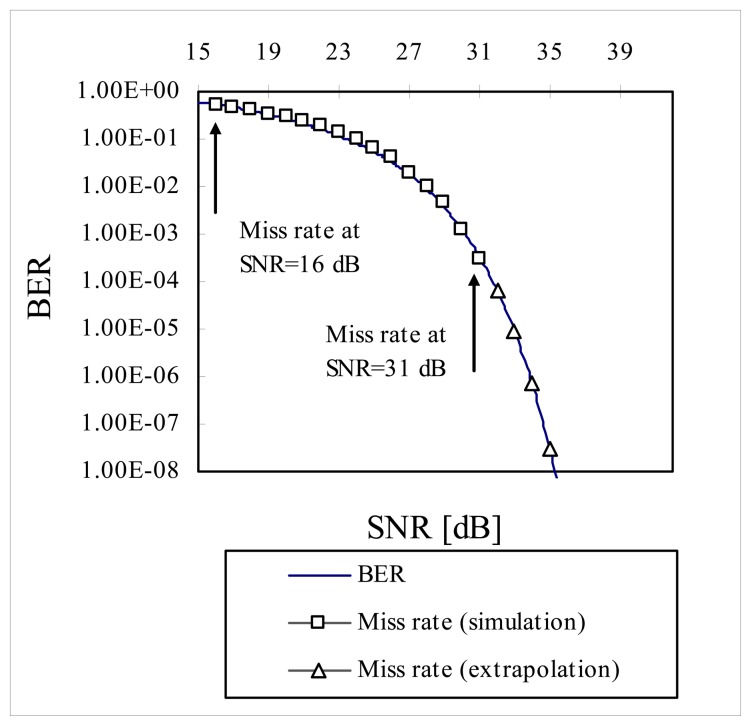
Extrapolation of miss rate on BER curve.

To extrapolate the miss rate beyond the range of 10^-4^ on the BER curve, data points in abscissa in Figure 9 were left-shifted by ∼14.2 dB (by trial and error) until data points of miss rate between 16 dB ∼ 31 dB were visually fitted on the BER curve. Then four more data points were extrapolated beyond the data point of miss rate at 31 dB *SNR* on the BER curve as shown in Figure 10. It is predicted that miss rate will be 3 × 10^-8^ at *SNR* of 35 dB.

### 4.2. Simulation: Root mean square (RMS) error of the zero order fringe peak identification

After the zero order fringe peak was identified, fine tuning was calculated for resolution enhancement. Phase error Φ*_error_*_,_*_i_* between computer generated zero order fringe peak *p_t_* (or *p*_0_) and fine-tuned zero order fringe peak *p̂_t_* (or *p̂_0_*) was calculated. Phase error Φ*_error_*_,_*_i_* was averaged over 30 simulations at a given *SNR* and this average gives out the root mean square (RMS) error of the zero order fringe peak identification.

(26) $$\Phi_{\text{RMS}}=\sqrt{\frac{\sum_{i=1}^{30}{\left(\Phi_{\text{error},i}\right)}^{2}}{30}}$$

**Figure 11.**
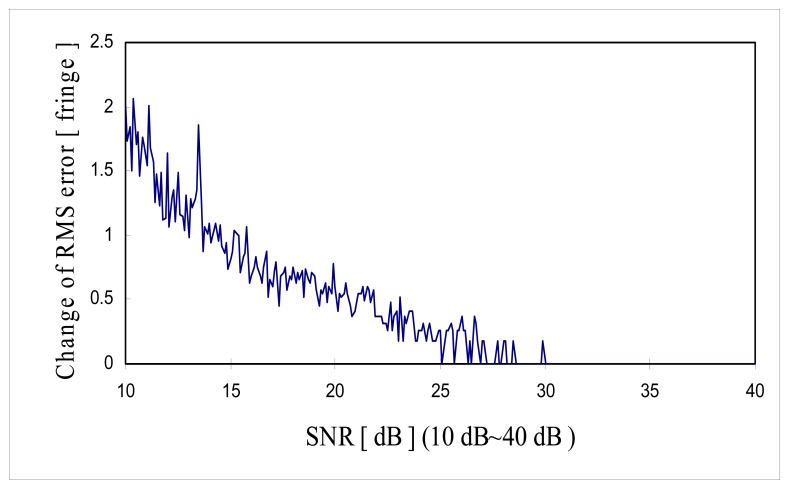
Change of RMS error as a function of *SNR* (10∼40 dB)

**Figure 12.**
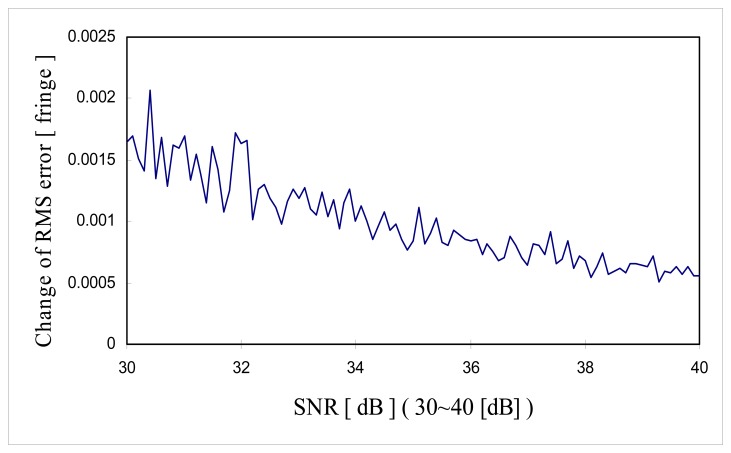
Change of RMS error as a function of *SNR* (30∼40 dB).

Figure 11 shows the change of RMS error along with the *SNR* in the range of 10 dB to 40 dB and Figure 12 shows the change of RMS error along with the *SNR* in the range of 30 dB to 40 dB. It is shown that the minimum *SNR* required to achieve RMS error less than 10^-3^ [fringe] (which is the fine tuning step size) must be greater than 35 dB *SNR*.

### 4.3. Simulation: High Precision Performance of Fine Tuning

In this simulation, the *SNR* was fixed at 30 dB and RMS error was compared between two cases. One is the case where the estimated zero order fringe peak *p̂_0_* was fine-tuned for resolution enhancement and the other case was where the estimated zero order fringe peak *p̂_0_* was not fine-tuned.

**Figure 13.**
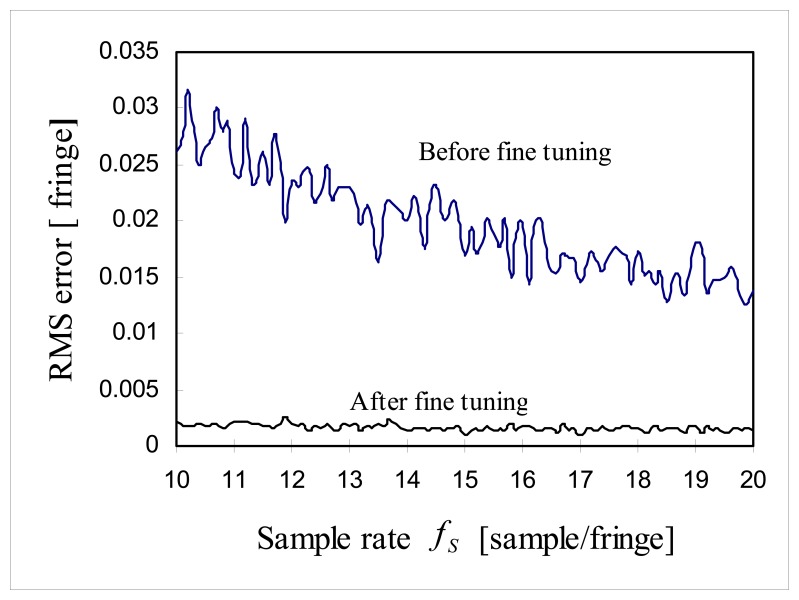
Comparison between RMS error with fine tuning and RMS error without fine tuning.

As shown in Figure 13, without fine tuning, RMS error of *p̂*_0_ is approximately 0.3/*f_S_* fringe and RMS error of *p̂*_0_ totally depends on the sample rate. But this RMS error was reduced down to ∼0.0015 fringe when *p̂*_0_ was fine tuned. Note that RMS error was not sensitive to the sample rate *f_S_* over the range between 10∼20 samples/fringe. This feature predicts that signal processing algorithm can use lower sample rate *f_S_* for the faster signal processing, but still maintain lower RMS error by using fine tuning, which is comparable to the error of higher sample rate *f_S_*. In the proposed algorithm, the cross-correlation between *i_S_*(*n*) and *i_R_*(*n*) is most time-consuming part of signal processing algorithm, but the sample rate *f_S_* can be lowered to speed up the signal processing without losing higher sample rate RMS error. One strong benefit of the proposed algorithm is that once zero order fringe is identified correctly then the resolution of the fine tuning can be adjusted as low as desired simply by changing the value of *N_sub_* and high precision phase delay estimation can be obtained.

### 4.4. Simulation: Effects of b̂ (f̂_S_)

**Figure 14.**
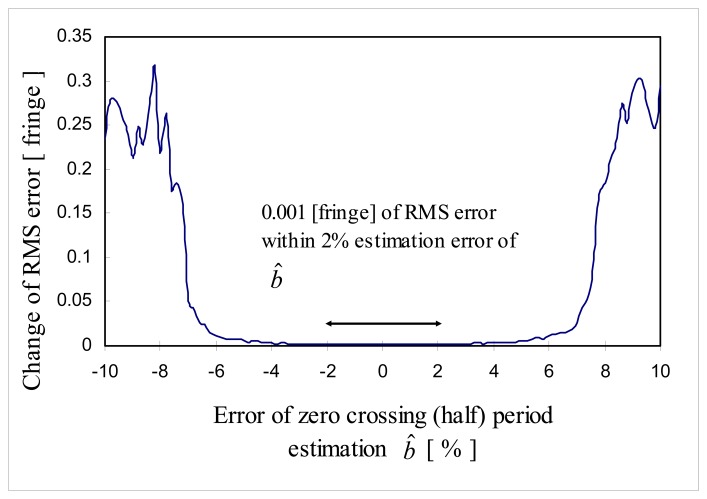
Change of RME error as a function of estimation error in zero crossing period.

In this simulation, estimated effective coherence length *L̂_C_*_,_*_eff_* was set as 36 fringes, *SNR*=30 dB and estimation error of the zero crossing period *b̂* was varied from -10% to 10% when *b*=10 (*f_S_* =20 samples/fringe) to see the effect of estimation error of the zero crossing period *b̂* on the performance of the proposed signal processing algorithm. The estimation error of the zero crossing period *b* is defined by:

(27) $$\text{Estimation error of}\;b=\frac{\hat{b}-b}{b}\times 100[\%]$$

where *b* is the half the sample rate *f_S_*. In Figure 14, it can be shown that RMS error of fine tuning was not sensitive to the estimation error of *b̂* within the range of ±6% estimation error of *b* and especially, RMS error of 0.001 [fringe] was obtained within the range of 2% estimation error of *b*. But, on the range of ±7% ∼ ±10% estimation error *b̂* RMS error increased dramatically to 0.25 fringe.

This is presumably due to the fact that *i*(*n*) and test cross-correlation *i_test_*(*n*) (Equation 24 or Equation 25) started to be out of phase and RMS error increased fast.

### 4.5. Simulation: Effects of L̂_C_

**Figure 15.**
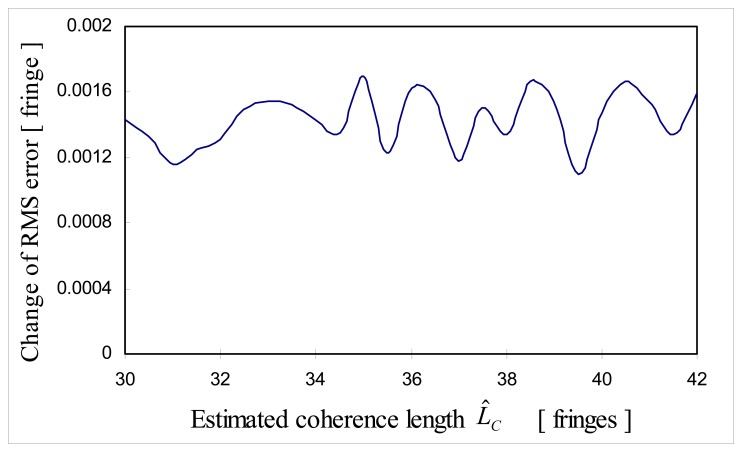
Change of RMS error as a function of estimation error in light source coherence length.

This simulation is to show the effect of estimated coherence length *L̂_C_* on RMS error assuming that *b̂* = *b, L_C_*=26λ(*L_C_*_,_*_eff_* ≈ 36λ) and *SNR* was fixed as 30 dB. Estimated coherence length *L̂_C_* was varied from 21λ to 30λ. In other words, estimated effective coherence length *L̂_C_*_,_*_eff_* was varied from 30λ to 42λ and RMS error was observed. Figure 15 shows that RMS error turned out to be not sensitive to the estimation error in coherence length *L̂_C,eff_*. This is due to the property of cross-correlation. Cross-correlation is maximized when *p_t_* and *p_d_* +*M*Δ*n* are in phase as long as both cross-correlation *i*(*n*) and test cross-correlation *i_test_* (*n*) are symmetric.

## 5. Comparison to the literature

In this section a comparison to the previous literature data regarding the resolution of zero order fringe peak detection is given. Interestingly enough, reference [20] calculated the theoretical limit of scanning white light interferometry signal evaluation algorithm. In this reference the theoretical limit of resolution of the fringe order detection was given as:

(28) $$\Delta z_{c}\approx \frac{N}{{\left[\frac{1}{\Delta z}\int_{-\infty }^{+\infty }I^{\prime }{\left(z\right)}^{2}dz\right]}^{1/2}}$$

(29) $$\begin{array}{cc} S_{i}=S(z_{i})=I(z_{i}-z_{c})+N(z_{i}), & i=0,\ldots ..n \end{array}$$

where *S_i_*=*S(z_i_)* consists of a fixed number of intensity values, typically taken at equidistant positions *z_i_*, *I*(*z*) is the ideal input signal (i.e. fringe scan) to be subject to intensity noise *N*(*z*), *z_c_* of *I*(*z*) is the position to be found using the evaluation algorithm (signal processing algorithm). And it is assumed that intensity noise *N*(*z*) is the constant noise value *N* over all samples. In our case, cross-correlation *i*(*n*) can be represented as the generalized equation as follows again:

(30) $$i(n)=1+I_{0}\exp\left[-4{\left(\frac{z}{l_{c,\text{eff}}}\right)}^{2}\right]\cos\left(\frac{2\pi n}{\lambda }\right)$$

Then, the derivative of *i*(*n*) becomes:

(31) $$i^{\prime }(z)=\left[\left(-8I_{0}\right){\left(\frac{1}{l_{c,\text{eff}}}\right)}^{2}\right]\;z\exp\left\{-4{\left[\frac{z}{l_{c,\text{eff}}}\right]}^{2}\right\}$$

and (*i*′(*z*))^2^ is given as:

(32) $$\begin{array}{cc} {\left(i^{\prime }(z)\right)}^{2}={\left[\left(-8I_{0}\right){\left(\frac{1}{l_{c,\text{eff}}}\right)}^{2}\right]}^{2} & z^{2}\exp\left\{-8{\left[\frac{z}{l_{c,\text{eff}}}\right]}^{2}\right\} \end{array}$$

Then, substituting Equation 32 into Equation 28 produces:

(33) $$\Delta z_{c}\approx \frac{N}{I_{0}}\times \sqrt{\Delta zl_{c,\text{eff}}}\times \frac{\sqrt{2}}{4}\times {\left(\frac{8}{\pi }\right)}^{\frac{1}{4}}=\frac{\sqrt{\Delta zl_{c,\text{eff}}}\times \frac{\sqrt{2}}{4}\times {\left(\frac{8}{\pi }\right)}^{\frac{1}{4}}}{I_{0}/N}$$

*I*_0_*/N* in denominator in Equation 33 is the signal-to-noise ratio and Equation 33 becomes:

(34) $$\Delta z_{c}\approx \frac{\sqrt{\Delta zl_{c,\text{eff}}}\times 0.353\times 1.263}{I_{0}/N}\lambda$$

As can be seen from the Equation 34, theoretical limit of fringe order detection is the function of sample rate, effective coherence length and *SNR*. Theoretical limit of resolution for the computer simulation shown in Figure 11 and Figure 12 can be calculated using the parameters of 16 sample per fringe (Δ*z*= λ/16), effective coherence length $l_{c,\text{eff}}=\sqrt{2}\times 26\times \lambda =36\lambda$. Then theoretical limit of resolution of zero order fringe detection in Figure 11 and Figure 12 is given as:

(35) $$\Delta z_{c}\approx \frac{\sqrt{\left(\lambda /16\right)\left(36\lambda \right)}\times 0.353\times 1.263}{\text{SNR}}=\frac{0.669\times \lambda }{\text{SNR}}(\text{without fine tuning})$$

(36) $$\Delta z_{c}\approx \frac{\sqrt{\left(\lambda /1000\right)\left(36\lambda \right)}\times 0.353\times 1.263}{\text{SNR}}=\frac{0.0846\times \lambda }{\text{SNR}}(\text{without fine tuning})$$

**Figure 16.**
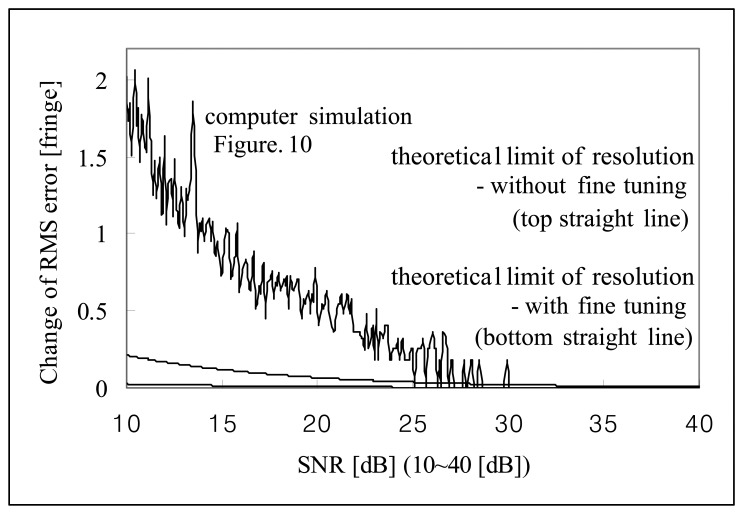
Comparison of computer simulation and theoretical limit (*SNR*: 10-40 [dB]).

**Figure 17.**
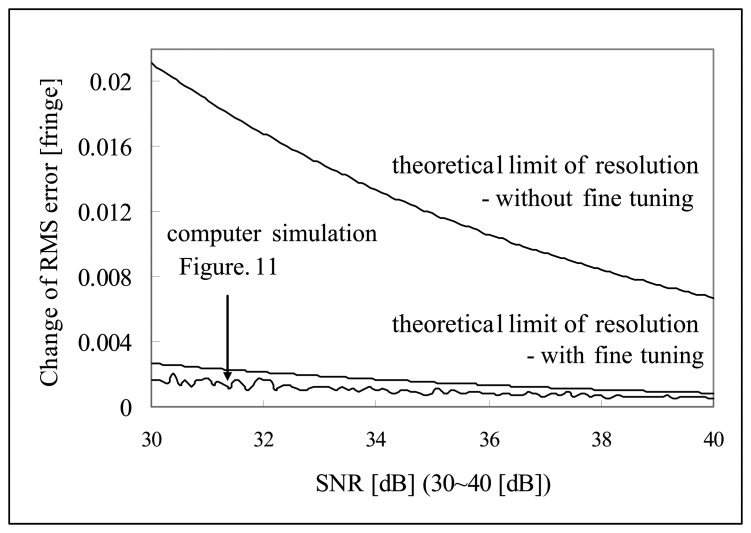
Comparison of computer simulation and theoretical limit (*SNR*: 30-40 [dB]).

Figures 16 and 17 are the comparison of the computer simulation results and the theoretical limit of fringe order detection calculated by Equation 35 and Equation 36. As shown in Figure 16, computer simulation results produce a much bigger RMS error than the theoretical limit over low *SNR* range (10∼30 [dB]). In comparison to the literature, it seems like that theoretical limit of reference [20] is more optimistic over low *SNR* range than the computer simulation performance. But, the proposed signal processing algorithm has proven to reach the theoretical limit of fringe order detection over the higher *SNR* range (30∼40 [dB]). This is not a surprising result because in higher *SNR* fine tuning algorithm will enhance the fringe order detection resolution down to the theoretical limit once zero order fringe peak is identified correctly. But, over low *SNR* range, fine tuning algorithm is not effective in enhancing the fringe order detection resolution because probably zero order fringe peak is misidentified and fine tuning is searched within the half sample of the misidentified zero order fringe peak.

Increasing the fine tuning range will help to locate the zero order fringe peak correctly and lower the RMS error down to the theoretical limit, although this is time consuming. Optimum fine tuning range of *M* over low *SNR* range to find the correct zero order fringe peak will be the subject of further research.

## 6. Conclusions

A new signal processing algorithm for white light interferometry has been proposed. The goal of signal processing algorithm was to find the time delay (phase shift) between two fringe scans which makes it possible to measure the absolute optical path length of a sensing interferometer. This new signal processing algorithm can be used for absolute temperature measure measurement by mapping the zero order fringe peak position of cross-correlation *i*(*n*) to the time delay between two fringe scan. Cross-correlation between two fringe scans utilizes all the photons in fringe scans effectively and the uncertainty of the zero order fringe peak mis-identification was reduced. Monte-Carlo simulations showed that the proposed signal processing algorithm identified the zero order fringe peak with a miss rate of 3×10^-4^ at 31 dB shot noise and the extrapolated miss rate at 35 dB shot noise was 3×10^-8^. Also resolution of less than 10^-3^ fringe was obtained at 35 dB shot noise (Figure 12). The fine tuning of signal processing algorithm requires some prior knowledge on the coherence length *L_C_* of the SLD and sample rate *f_S_*. But the performance of proposed signal processing algorithm turned out to be not sensitive to the estimation error in *L̂_C_* and *f̂_S_* (within the range from −6% to 6% estimation error of *b̂*). Also the proposed signal processing reached the theoretical limit of fringe order detection over the higher *SNR* range (30∼40 [dB]). The proposed signal processing algorithm uses a software approach which is potentially inexpensive, simple and fast. As a whole, the proposed signal processing algorithm has proven to be a high precision signal processing algorithm for AFWLI phase (time) delay estimation.

## Figures and Tables

**Figure 6.**
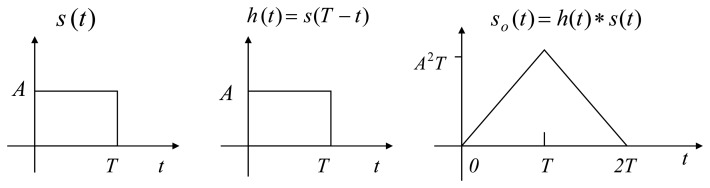
Matched filtering.

**Table 1.** Example of peak and zero crossing table (*f_S_* =20 [samples/fringe]).

Peak and zero crossing label	*p*_0_	*z*_100_	*q*_1_	*z*_101_	*p*_1_
Position	35	39.34	45	50.13	55

**Table 2.** Miss rate of the proposed signal processing algorithm as a function of *SNR*.

***SNR* [dB]**	**Miss rate**	***SNR* [dB]**	**Miss rate**

1	0.95	17	0.46
2	0.94	18	0.40
3	0.93	19	0.34
4	0.91	20	0.30
5	0.89	21	0.23
6	0.87	22	0.19
7	0.85	23	0.14
8	0.83	24	0.10
9	0.80	25	0.066
10	0.76	26	0.041
11	0.73	27	0.019
12	0.69	28	0.009
13	0.64	29	0.004
14	0.60	30	0.001
15	0.56	31	0.0003
16	0.51	32	0

## Appendix A

In this appendix, *SNR* improvement at the peak of cross-correlation *i*(*t*) is derived. First, *i_S_* (Φ) and *i_R_*(Φ) are given respectively as:

(A-1) $$i_{S}(\Phi_{P})=\exp\left\{-{\left[\frac{\Phi_{P}-\Phi_{S}}{\pi L_{C}/\lambda }\right]}^{2}\right\}\cos(\Phi_{P}-\Phi_{S})$$

(A-2) $$i_{R}(\Phi_{P})=\exp\left\{-{\left[\frac{\Phi_{P}-\Phi_{R}}{\pi L_{C}/\lambda }\right]}^{2}\right\}\cos(\Phi_{P}-\Phi_{R})$$

where $\Phi_{P}=\frac{2\pi nL_{P}}{\lambda }$, $\Phi_{S}=\frac{2\pi nL_{S}}{\lambda }=\Phi_{R}+\Phi_{d}$, $\Phi_{R}=\frac{2\pi nL_{R}}{\lambda }$

Here *L_P_*, *L_S_*, *L_R_* are the path length difference of the Mach-Zehnder processing interferometer, sensing FFPI (twice the Fabry-Perot cavity length), reference FFPI and *n* is the effective refractive index of core of the optical fiber (not to be confused with sample number *n* in *i*(*n*)). Notation ⨂,* and ⇔ are reserved to denote the cross-correlation, convolution and Fourier transform relationship.

When *L_P_* is scanned *L_P_* is a function of time *t* and also Φ*_P_* is a function of time *t*. Then:

(A-3) $$\Phi_{P}=2\pi ft=w_{0}t$$

where *f* (or *w*_0_) is a modulation frequency (or angular velocity) of fringe scan. Following the same token,Φ*_S_*, Φ*_R_* are given by:

(A-4) $$\Phi_{S}=2\pi ft_{S}=w_{0}t_{S}$$

(A-5) $$\Phi_{R}=2\pi ft_{R}=w_{0}t_{R}$$

and the relation between *t_S_* and *t_R_* is given by:

(A-6) $$t_{S}=t_{R}+t_{d}$$

where *t_d_* is the time delay between sensing FFPI fringe scan and reference FFPI fringe scan.

Using the following substitutions:

(A-7) $$\Phi_{P}-\Phi_{S}=w_{0}(t-t_{S})=w_{0}(t-t_{R}-t_{d})$$

(A-8) $$\Phi_{P}-\Phi_{R}=w_{0}(t-t_{R})$$

(A-9) $$L_{C}=N_{f}\lambda$$

and considering the noise in *i_S_*(Φ*_P_*) and *i_R_*(Φ*_P_*), time domain representation of the Equation A-1 and Equation A-2 can be rewritten as:

(A-10) $$i_{S}(t)=e^{-a{\left(t-t_{d}\right)}^{2}}\cos(w_{0}(t-t_{d}))+w_{S}(t)$$

(A-11) $$i_{R}(t)=e^{-at^{2}}\cos(w_{0}(t))+w_{R}(t)$$

In Equation A-10 and A-11 we used a substitution *a* = *(w*_0_/*πN_f_*)^2^ and it is assumed that Φ*_R_*=0 without loss of generality and that *w_S_*(*t*), *w_R_*(*t*) are white noise with zero mean and variance $\sigma_{N}^{2}$ of *i_S_*(*t*), i_R_(*t*) respectively and independent each other. Also, *w*_0_ was set to “1” for normalization in order to make *i_S_*(*t*) = *e^-^*^1^ at *t* = (*N_f_*/2) × 2π.

The first part of Appendix A shows that the increase of coherence length from *L_C_* to $\sqrt{2}L_{C}$ due to cross-correlation. We know that cross-correlation and convolution are identical operations for the case where either *i_S_*(*t*) or *i_R_* (*t*) is an even symmetric function. Then, the cross-correlation *i*(*t*) of *i_S_*(*t*) and *i_R_*(*t*) is given as follows:

(A-12) $$\begin{array}{c} i_{S}(t)⊗i_{R}(t)=i_{S}(t)*i_{R}(t)=(e^{-a{\left(t-t_{d}\right)}^{2}}\cos(t-t_{d})+w_{S}(t))*(e^{-at^{2}}\cos t+w_{R}(t))\\ =\int_{-\infty }^{+\infty }(e^{-a\lambda^{2}}\cos\lambda +w_{R}(\lambda ))(e^{-a{\left(t-\lambda -t_{d}\right)}^{2}}\cos(t-\lambda -t_{d})+w_{S}(t-\lambda ))d\lambda \\ =\int_{-\infty }^{+\infty }((e^{-a\lambda^{2}}\cos\lambda )(e^{-a{\left(t-\lambda -t_{d}\right)}^{2}}\cos(t-\lambda -t_{d}))d\lambda +\int_{-\infty }^{+\infty }(e^{-a\lambda^{2}}\cos\lambda )(w_{S}(t-\lambda ))d\lambda \\ \int_{-\infty }^{+\infty }(w_{R}(\lambda ))(e^{-a{\left(t-\lambda -t_{d}\right)}^{2}}\cos(t-\lambda -t_{d}))d\lambda +\int_{-\infty }^{+\infty }w_{R}(\lambda )w_{S}(t-\lambda ))d\lambda \end{array}$$

The first term in Equation A-12 is the cross-correlation of noise free interference signal *i_NF_*(*t*) = *e*^−^^*at*^2^^ cos(*t*) and its delayed version *i_NF_*(*t* − *t_d_*) = e^−(^*^t − td^*^)2^ cos(*t* − *t_d_*), which is calculated as follows:

(A-13) $$i_{NF}(t)⊗i_{NF}(t-t_{d})=i_{NF}(t)*i_{NF}(t-t_{d})=\left(e^{-at^{2}}\cos t\right)*\left(e^{-a{\left(t-t_{d}\right)}^{2}}\cos(t-t_{d})\right)$$

Well-known Fourier transform relationships useful for the calculation of Equation A-13 are:

(A-14) A(t)∗B(t)⇔A(w)B(w)

(A-15) $$A(t)B(t)\Leftrightarrow \frac{1}{2\pi }A(w)*B(w)$$

(A-16) $$\frac{1}{\left|\alpha \right|}A\left(\frac{t}{\alpha }\right)\Leftrightarrow A(\alpha w)$$

where:

(A-17) A(t)⇔A(w)andB(t)⇔B(w)

and *w* is the frequency domain variable.

If we only consider the envelope of *i_NF_*(*t*) = *e^−at2^* cos*t*, i.e. *i_NF_*_,_*_env_* (*t*) = *e*^−^^*at*^2^^ and use the Fourier transform relation:

(A-18) $$e^{-at^{2}}\Leftrightarrow \sqrt{\frac{\pi }{a}}e^{-\frac{w^{2}}{4a}}$$

then Fourier transform of Equation A-13 becomes:

(A-19) $$i_{NF,\text{env}}(t)*i_{NF,\text{env}}(t-t_{d})=\left(e^{-at^{2}}\right)*\left(e^{-a{\left(t-t_{d}\right)}^{2}}\right)\Leftrightarrow (\sqrt{\frac{\pi }{a}}e^{\frac{w^{2}}{4a}}\sqrt{\frac{\pi }{a}}e^{\frac{w^{2}}{4a}})e^{-jwt_{d}}=(\sqrt{\frac{\pi }{a}}\sqrt{\frac{\pi }{a}}e^{\frac{{\left(\sqrt{2}w\right)}^{2}}{4a}})e^{-jwt_{d}}$$

Then *i_NF_*_,_*_env_*(*t*)**i_NF_*_,_*_env_*(*t−t_d_*) is the inverse Fourier transform of $(\sqrt{\frac{\pi }{a}}\sqrt{\frac{\pi }{a}}e^{-\frac{{\left(\sqrt{2}w\right)}^{2}}{4a}})e^{-jwt_{d}}$, which is given as:

(A-20) $$i_{NF,\text{env}}(t)*i_{NF,\text{env}}(t-t_{d})=\sqrt{\frac{\pi }{a}}\frac{1}{\sqrt{2}}e^{-a{\left(\frac{t-t_{d}}{\sqrt{2}}\right)}^{2}}$$

Before cross-correlation, from Equation A-10 and Equation A-11, peak value of *i_S_*(*t*) is 1 and *i_S_*(*t*) reduces down to *e^−^*^1^ of its peak value at:

(A-21) $$t=\pm \frac{1}{\sqrt{a}}(\text{for Equation A}-10)\;\text{and}\;t=t_{d}\pm \frac{1}{\sqrt{a}}(\text{for Equation A}-11)$$

After cross-correlation, from Equation A-20,*e^-^*^1^ of *i_S_* ⨂ *i_s_*(*t*) happens at:

(A-22) $$t=t_{d}\pm \frac{\sqrt{2}}{\sqrt{a}}$$

And the coherence length of the cross-correlation (effective coherence length) has increased by a factor of $\sqrt{2}$. In other words, $L_{C,\text{eff}}=\sqrt{2}L_{C}$

The second part of appendix A shows that 14 dB *SNR* improvement is obtained at zero order fringe peak after cross-correlation. Peak value (zero-order fringe peak) of cross-correlation *i*(*t*) happens when *t* = *t_d_* and its value *i_S_** *i_R_* (*t*=*t_d_*) is calculated by substituting *t* = *t_d_* in Equation A-12 as follows:

(A-23) $$\begin{array}{l} i_{s}*i_{R}(t_{d})=\int_{-\infty }^{+\infty }((e^{-a\lambda^{2}}\cos\lambda )(e^{-a{\left(-\lambda \right)}^{2}}\cos(-\lambda ))d\lambda +\int_{-\infty }^{+\infty }\left(e^{-a\lambda^{2}}\cos\lambda \right)(w_{s}(t_{d}-\lambda ))d\lambda \\ +\int_{-\infty }^{+\infty }\left(w_{R}(\lambda )\right)(e^{-a{\left(-\lambda \right)}^{2}}\cos(-\lambda ))d\lambda +\int_{-\infty }^{+\infty }w_{R}(\lambda )w_{s}(t_{d}-\lambda ))d\lambda \end{array}$$

The first term of Equation A-23 is the signal component which is the energy of noise free interference signal *i_NF_* (*t*) = *e^−at^*^2^ cos(*t*) and the other three terms of Equation A-23 are noise terms. Then the variance of three noise terms are given as:

(A-24) $$(2\int_{-\infty }^{+\infty }{\left(e^{-a\lambda^{2}}\cos\lambda \right)}^{2}\;d\lambda )\times \sigma_{N}^{2}+(\int_{-\infty }^{+\infty }w_{R}{\left(\lambda \right)}^{2}d\lambda )\times \sigma_{N}^{2}$$

Then *SNR* at the peak of cross-correlation *i*(*t*) is given as the ratio between magnitude of peak signal to the standard deviation of noise as shown in Equation A-25.

(A-25) $$\text{SNR}=\frac{\int_{-\infty }^{+\infty }{\left(e^{-a\lambda^{2}}\cos\lambda \right)}^{2}d\lambda }{\sqrt{2\int_{-\infty }^{+\infty }{\left(e^{-a\lambda^{2}}\cos\lambda \right)}^{2}d\lambda )\sigma_{N}^{2}+(\int_{-\infty }^{+\infty }w_{R}{\left(\lambda \right)}^{2}d\lambda )\sigma_{N}^{2}}}$$

By assuming that the variance of the noise is much smaller than the power of signal:

(A-26) $$\int_{-\infty }^{+\infty }{\left(e^{-a\lambda^{2}}\cos\lambda \right)}^{2}d\lambda \gg \int_{-\infty }^{+\infty }w_{R}{\left(\lambda \right)}^{2}d\lambda$$

Equation A-25 can be approximated to:

(A-27) $$\text{SNR}\approx \frac{\int_{-\infty }^{+\infty }{\left(e^{-a\lambda^{2}}\cos\lambda \right)}^{2}d\lambda }{\sqrt{(2\int_{-\infty }^{+\infty }{\left(e^{-a\lambda^{2}}\cos\lambda \right)}^{2}d\lambda )\sigma_{N}^{2}}}=\frac{\sqrt{\int_{-\infty }^{+\infty }{\left(e^{-a\lambda^{2}}\cos\lambda \right)}^{2}d\lambda }}{\sqrt{2}\sigma_{N}}$$

Again, *SNR* of *i_S_*(*t*) (or *i_R_*(*t*)) before the cross-correlation is:

(A-28) $$\text{SNR}=\frac{i_{NF,\text{MAX}}}{\sigma_{N}}$$

so cross-correlation has improved the *SNR* by less than a factor of:

(A-29) $${\text{SNR}}_{\text{improve}}=\frac{\sqrt{\int_{-\infty }^{+\infty }{\left(e^{-a\lambda^{2}}\cos\lambda \right)}^{2}d\lambda }}{\sqrt{2}\sigma_{N}}\times \frac{\sigma_{N}}{i_{NF,\text{MAX}}}=\frac{1}{\sqrt{2}}\frac{\sqrt{\int_{-\infty }^{+\infty }e^{-2a\lambda^{2}}\cos^{2}\lambda d\lambda }}{i_{NF,\text{MAX}}}$$

It can be shown that energy of the (noise-free) interference signal $\int_{-\infty }^{+\infty }e^{-2a\lambda^{2}}\cos^{2}\lambda \;d\lambda$ in Equation A-29 is given by $\frac{1}{2}\frac{1}{\sqrt{2}}\sqrt{\frac{\pi }{a}}=\frac{1}{2}\frac{1}{\sqrt{2}}\sqrt{\pi^{3}N_{f}^{2}}$ as follows:

(A-30) $$i_{NF}*i_{NF}(0)=\int_{-\infty }^{+\infty }e^{-2ax^{2}}\cos^{2}\text{xdx}=\frac{1}{2}\int_{-\infty }^{+\infty }e^{-2ax^{2}}(1+\cos2x)dx$$

Useful relations are given by:

(A-31) $$\int_{-\infty }^{+\infty }e^{-2ax^{2}}dx=\frac{1}{\sqrt{2}}\sqrt{\frac{\pi }{a}}$$

(A-32) $$\int_{-\infty }^{+\infty }e^{-2ax^{2}}\cos2\text{xdx}=\frac{1}{\sqrt{2}}\sqrt{\frac{\pi }{a}}e^{\frac{1}{2a}}$$

Then Equation A-30 becomes:

(A-33) $${\int_{-\infty }^{+\infty }e^{-2ax}\cos^{2}x\;dx=\frac{1}{2}\frac{1}{\sqrt{2}}\sqrt{\frac{\pi }{a}}+\frac{1}{2}\frac{1}{\sqrt{2}}\sqrt{\frac{\pi }{a}}e}^{-\frac{1}{2a}}$$

But, the second term in Equation A-29 is negligible because $e^{-\frac{1}{2a}}$ has a negligible value and Equation A-33 becomes:

(A-34) $$\int_{-\infty }^{+\infty }e^{-2ax^{2}}\cos^{2}x\;dx=\frac{1}{2}\frac{1}{\sqrt{2}}\sqrt{\frac{\pi }{a}}=\frac{1}{2}\frac{1}{\sqrt{2}}\sqrt{\frac{\pi }{a}}\sqrt{\pi^{3}N_{f}^{2}}$$

Then, finally *i_NF_*_,_*_MAX_* is “1” and combining Equation A-29 and Equation A-34 we get:

(A-35) $$20\log({\text{SNR}}_{\text{improve}})=20\log(\frac{\sqrt{\int_{-\infty }^{+\infty }{\left(e^{-2a\lambda^{2}}\cos\lambda \right)}^{2}d\lambda }}{2})=20\log(\frac{\sqrt{\frac{1}{2}\frac{1}{\sqrt{2}}\sqrt{\pi^{3}N_{f}^{2}}}}{\sqrt{2}})$$

which is 14 dB when *N_f_* = 26
